# Telemedicine Teaching for Everyone? Comparing Responses of Medical Students and Residents with a Shared Curriculum

**DOI:** 10.1089/tmr.2024.0070

**Published:** 2025-04-11

**Authors:** Kristi VanDerKolk, Shamsi Daneshvari Berry, Lisa Graves

**Affiliations:** ^1^ Department of Family and Community Medicine, Western Michigan University Homer Stryker M.D. School of Medicine, Kalamazoo, Michigan, USA.; ^2^ Department of Biomedical Informatics, Western Michigan University Homer Stryker M.D. School of Medicine, Kalamazoo, Michigan, USA.

**Keywords:** health care delivery/health services research, education and/or curriculum development, graduate medical education, undergraduate medical education

## Abstract

**Introduction:**

The COVID-19 pandemic forced rapid increases in the use of telemedicine, which requires a different skillset than in-person visits. Both medical students and residents required urgent training in telemedicine to utilize the technology for patient care. This study compares evaluation outcomes using the same shared national curriculum for medical students and family medicine residents at the same institution.

**Methods:**

Medical students and family medicine residents at Western Michigan University Homer Stryker M.D. School of Medicine completed and evaluated the Society of Teachers of Family Medicine Telemedicine Task Force national curriculum on best practices and foundations within the realm of telemedicine. A Mann–Whitney analysis was performed on Likert scale questions.

**Results:**

Medical students had significantly greater knowledge, skill, or attitude acquisition from Module 1, the basics of telemedicine, than family medicine residents. There were no significant differences in knowledge, skill, or attitude acquisition in Module 2, 3, 4, or 5.

**Discussion:**

The results from this single-institution sample of medical students and family medicine residents using a shared telemedicine curriculum illustrate important differences from the national data. Unlike the national data, there was a significant difference in knowledge, skill, or attitude acquisition in Module 1, while there was no difference in knowledge, skill, or attitude acquisition in the remaining four modules. Our results indicate changing utility of the telemedicine modules based on previous and ongoing experience. Learners of multiple levels find utility in a shared curriculum in teaching novel concepts.

## Introduction 

The COVID-19 pandemic forced health care systems to evolve rapidly. Telemedicine (clinical) and telehealth (both clinical and non-clinical) were used as physicians, and their patients sought to safely participate in medical care.^1^ In a 2021 American Medical Association survey, 85% of physicians self-reported current telemedicine use.^2^ Studies show high patient satisfaction with telemedicine visits.^3–5^ Patients have indicated a preference for continued access to telemedicine services post pandemic.^6^

With the rapid rise in telehealth, undergraduate and graduate medical education leaders have recognized the need for training in its appropriate, effective, and efficient use.^7^ In Association of American Medical Colleges (AAMC) surveys of medical schools in the years prior to the pandemic, less than half included telehealth in their curriculum (2015–2017 academic years). This escalated to over 80% in academic year 2020.^8^ The Family Medicine (FM) Residency Review Committee’s 2023 update on core competencies now includes several references to the use of telehealth. FM residency practices must have telehealth modalities available, and residents must show competence in telehealth use by graduation.^9^

Telemedicine’s necessarily rapid uptake occurred without significant consideration of clinical education needs.^10^ Clinical education leaders quickly realized the need for teaching its use to residents and medical students.^7,8,11,12^ Early surveys indicated the utilization of didactic sessions and clinical experience for teaching medical students about telemedicine.^10^ Educational efforts were complicated by availability of minimal educational materials and limited faculty expertise.^7,8,11,12^ The Society of Teachers of Family Medicine (STFM) Telemedicine Task Force developed a national curriculum focused on best practices and foundations in telemedicine. This curriculum was specifically aimed at medical students and FM residents and was based on the AAMC telehealth competencies. Additional competencies were added based on committee consensus. Importantly, the curriculum was designed to be provided asynchronously via an interactive virtual platform.^7^ This design, therefore, differs from the previously described didactic or clinical settings for education.^10^ The curriculum was piloted at medical schools and FM residencies throughout North America and noted to be broadly effective. The curriculum consisted of five modules: 1—Intro to telehealth; 2—The telehealth encounter; 3—Requirements for telehealth; 4—Access and equity in telehealth; and 5—Future of telehealth.^7^ This study looks at a subset of the pilot evaluation outcomes for medical students and FM residents within a single institution to assess for efficacy in both groups of learners while minimizing variability in institutional differences in education, experience, and practice patterns.

## Materials and Methods

The STFM Telemedicine Task Force spent 1 year developing a telehealth curriculum utilizing the AAMC telehealth competencies and development group expertise. A detailed description of the process and curriculum has been previously published.^7^ The five modules applied Bloom’s taxonomy levels of apply, analyze, and synthesize and involve interactive exercises and case-based decision-making.^13^ Seventeen medical schools and 17 FM residency programs piloted the curriculum. Only one institution had both the medical school and a residency program selected, Western Michigan University Homer Stryker M.D. School of Medicine (WMed). The AAFP Institutional Review Board determined this study to be exempt and not human subjects’ research.

### Population studied

The single-institution sample consisted of 30 medical students and 40 residents (Table 1). Participating medical students were primarily in their third year of training, when they typically rotated through FM within this institution. Few students in the first and second years were included due to the completion of short clinical experiences in FM during this time frame. No fourth year medical students participated in the study as they work with FM in the inpatient as opposed to the outpatient setting. Residents from across the U.S. training duration of 3 years were included. Learners evaluated each module using both closed- and open-ended questions. Closed-ended questions included a 5-point Likert scale that included strongly disagree, disagree, neutral, agree, and strongly agree.

**Table 1.** Participants by Year in Program

Program	Year 1	Year 2	Year 3	Total
Undergraduate	1	1	28	30
Residency	15	13	12	40

### Statistical analysis

For statistical analysis, medical students at all training levels were combined into a single group and compared with a single group of FM residents at all training levels. The decision to add a single first-year and second-year medical student into the analysis group was made, given statistical analysis was unchanged with their inclusion. A Mann–Whitney analysis (*α* = 0.05) was conducted on both medical student and resident acquisition of knowledge, skills, or attitudes to improve clinical practice and the utility for a learner at their level of training. The analysis was conducted within each module separately. Answers of highly agree and agree as well as highly disagree and disagree were combined in the analyses. Therefore, the analyses compared the results of agree, neutral, and disagree.

Open-ended comments about each module were collected in response to the prompt: “what could STFM do to improve this module?” (Table 2). Following in-depth reviews of all comments, they were grouped into five thematic categories for analysis: no suggestions, shorten, improve interactivity, increase depth, and other. Thematic categorization was determined by one of the investigators (S.D.B.) and reviewed and agreed upon by the remaining investigators. Mann–Whitney tests (*α* = 0.05) were run to test the association between the comment and learner level by module.

**Table 2.** Comments from Open-Ended Question—“What Could STFM Do to Improve This Module?”

Module	Student level	Comment grouping	Comments included
1	Resident	No suggestions	N/A, nothing, its fine, no suggestion, its good, no comments, great module, nothing comes to mind, idk
		Shorten	
		Improve interactivity	More videos, video, more interaction, video/visuals, accompanying videos, incorporate sample/simulated video visits, make it shorter and more interactive, add video
		Increase depth	Include examples, more pictures, audio, video supplementation of good and bad interactions—how to overcome barriers, may be use more tables and illustrations
		Other	Make it faster, improve redundancy of this survey
	Medical student	No suggestions	NA, none, nothing, can’t think of anything, its well done, I like how there were accompanying videos for this module, not sure, well done
		Shorten	
		Improve interactivity	More interaction would make the information more memorable, more interaction, interaction, videos
		Increase depth	More examples of appropriate uses
		Other	Summary at the end, keep updating as evidence comes in
2	Resident	No suggestions	Well done, none, nothing, N/A, it’s well designed, I like how there were accompanying videos for this module, not sure
		Shorten	Make it shorter, videos were redundant, shorten videos
		Improve interactivity	Interactive, use real-life examples, more videos, more interactive, more visualization
		Increase depth	More emphasis on physical exam, tailor to residents—some very basic info included
		Other	Summary page
	Medical student	No suggestions	N/A, nothing, none, nothing it was great
		Shorten	Probably make it a little shorter, less long videos or allowing a 2× speed function, shorten it
		Improve interactivity	More videos, interactive visit, more virtual PE ideas
		Increase depth	
		Other	Provide video transcript, make it faster
3	Resident	No suggestions	N/A, none, nothing, nothing comes to mind, its good, good module, all good, no comment
		Shorten	
		Improve interactivity	More tables, more visualization, more pictures
		Increase depth	Real-life examples, nothing—the module itself was good for someone unfamiliar with televisits, but as someone who has been doing them all year, this is repeat information
		Other	More troubleshooting info, more questions, the evaluation questions didn’t all correlate with the available material, good for those with little computer experience otherwise not very helpful, summary page
	Medical student	No suggestions	N/A, none, no comment, not applicable, I thought the length was good
		Shorten	Shorter
		improve interactivity	Video, more downloadable note sample statements
		Increase depth	More relevant information, make information more relevant, provide more information on peripherals how they can be used and incorporated into a telemedicine encounter
		Other	Instead of linking to the different organizations, include brief overviews of general tips providers should keep in mind for telehealth visits
4	Resident	No suggestions	Nothing, none, well done, no comment, “-,” not sure
		Shorten	
		Improve interactivity	Graphics, more videos, more videos of the interactions
		Increase depth	More examples
		Other	Summary page
	Medical student	No suggestions	N/A, none, nothing, this was a really good one
		Shorten	Shorter
		Improve interactivity	Videos, simulations with actual encounters might be nice
		Increase depth	Tailor info to year of training, more material regarding crisis usage, more with special needs patients
		Other	Downloadable summary, this is a telehealth module—only include what is relevant to telehealth
5	Resident	No suggestions	N/A, none, nothing, no comment, “-,” nothing comes to mind
		Shorten	
		Improve interactivity	Videos, videos/graphics, more videos, more interactions
		Increase depth	More examples, more info about different tools in telemed tool boxes
		Other	Everything, not very helpful—consider removing, not really implementable in current residency practice—could touch more on the accuracy and feasibility of implementation of some of the remote devices (how well do noises transmit with electronic/remote stethoscopes, can patients place them appropriately on chest/back? etc.), summary page
	Medical student	No suggestions	N/A, no comment, none, nothing
		Shorten	Shorter
		Improve interactivity	Videos, maybe more videos demoing the tech rather than a lot of words about it
		Increase depth	Talk about drawbacks in fast advancements, provide links/access to more resources on the topics discussed
		Other	Downloadable summary, realistic skepticism on the future of telehealth—a lot of telehealth ideas sound like sales pitches, you need to consider what would incentivize use and will the use be as intended or desired. Many ideas of what telehealth could be are intriguing but lack the necessity or incentive from both providers and patients to make them happen

## Results

Comparative analyses of student and resident evaluations of the modules were significantly different in just two areas (Table 3). Student assessment of the level of knowledge gained in Module 1 was significantly greater than resident assessment (*p* = 0.0292). There was no significant difference in ratings on the level of knowledge gained between students and residents for the four remaining modules. There was a significant difference in student and resident open-ended comments on Module 4, with more residents than students commenting that the module was right on target. There was no significant difference in student and resident responses to categorical questions on knowledge gained and the level of the module.

**Table 3.** Student and Resident Evaluation of Modules

	Module 1	Module 2	Module 3	Module 4	Module 5
I gained new knowledge, skills, and/or attitudes	MS: 83 R: 60 (0.029^*^)	MS: 56 R: 73 (0.134)	MS: 58 R: 61 (0.771)	MS: 71 R: 62 (0.454)	MS: 67 R: 60 (0.577)
Information useful at my level of training	MS: 90 R: 90 (1)	MS: 84 R: 90 (0.88)	MS: 71 R: 78 (0.421)	MS: 79 R: 86 (0.453)	MS: 95 R: 88 (0.785)
Comments (no changes)	MS: 77 R: 60 (0.170)	MS: 68 R: 68 (0.987)	MS: 71 R: 76 (0.853)	MS: 67 R: 88 (0.038^*^)	MS: 67 R: 75 (0.519)
*N*	70	66	65	66	61

Percentage of agreement by medical students (MS) and residents (R) by module.

^*^ *p*-value (*p*) significant at *p* ≤ 0.05.

## Discussion

Medical students and residents have had limited exposure to telemedicine teaching.^14^ This pilot of a shared STFM curriculum in medical students and FM residents at a single institution demonstrated a significantly greater knowledge, skill, or attitude acquisition in medical students versus FM residents in Module 1. There was no significant difference between medical student and resident knowledge, skill, or attitude acquisition in any of the other modules. This is in contrast to national data that observed significantly greater agreement with knowledge, skill, or attitude acquisition of medical students versus FM residents in Modules 2, 3, 4, and 5, but not on Module 1, where there was no difference between the two groups.

Many of the WMed FM residents participated in rapid uptake of telemedicine early in the pandemic, in advance of most medical student exposure and prior to curricular exposure. Given their first-hand experience with telemedicine, Module 1, which focuses on appropriate uses of telemedicine along with its benefits and limitations, was likely already intuitive to the resident group.

Our expectation is that over the next several years, most residents will have received teaching on the basics of telemedicine as part of their undergraduate curriculum, leading to less need for introductory education in residency. WMed FM residents received little explicit instruction in the performance of telemedicine visits (Module 2), technological requirements and legal considerations of telemedicine (Module 3), the impact of telemedicine on health care access and equity (Module 4), and the future of telemedicine (Module 5) prior to its clinical use. Therefore, within the WMed institution, Modules 2, 3, 4, and 5 represented new information to both FM residents and medical students. Additionally, WMed FM resident interest in these modules may have been positively affected by their ongoing participation in telemedicine visits with resulting need and desire to improve skills in its use. With expanded teaching and utilization of telemedicine in undergraduate education, we may see a decline in resident satisfaction of the knowledge gained. This would be more similar to the national data, though it is unclear whether national data indicate increased knowledge or a lower perception of utility in FM residents versus medical students in Modules 2–5.

Medical education is a continuum from an inexperienced learner to a master clinician.^15^ This pilot suggests that in relatively novel areas of medical education, curriculum can be developed and used for multiple levels of learners. Intentional use by learners allows the opportunity for learning in a spiral manner, with participant’s ability to revisit areas of weakness. With continued use of telehealth, expansion of telemedicine education to include higher-level competencies, such as incorporation of physical exam, is needed.

While this study was limited by location within a single site, this limitation was leveraged to control for outside variables, allowing for consistency in the analysis between the two learner groups. Evaluation was limited to acquisition in knowledge, skill, and attitude, as well as the level of instruction. In future studies, we would include a faculty group as part of the learning continuum.

Knowledge of efficacy of a shared curriculum for both residents and medical students is a critical message for faculty looking to deploy innovative practices. We postulate that curriculum development can focus less on the stage of the learner and more on previous exposure.

## Abbreviation Used

AAFP: American Academy of Family Physicians
AAMC: Association of American Medical Colleges
FM: Family Medicine
STFM: Society of Teachers of Family Medicine
WMed: Western Michigan University Homer Stryker M.D. School of Medicine

## References

[1] Association AM. Policy Research Perspectives: Changes in Medicare physician spending during the COVID-19 pandemic. 2021;2019.

[2] Medical Association A. 2021 Telehealth Survey Report, AMA. 2022.

[3] Drerup B, Espenschied J, Wiedemer J, et al. Reduced no-show rates and sustained patient satisfaction of telehealth during the COVID-19 pandemic. Telemed J E Health 2021;27(12):1409–1415; doi: 10.1089/tmj.2021.0002 33661708

[4] Kruse CS, Krowski N, Rodriguez B, et al. Telehealth and patient satisfaction: A systematic review and narrative analysis. BMJ Open 2017;7(8):e016242. https://pubmed.ncbi.nlm.nih.gov/28775188/10.1136/bmjopen-2017-016242 PMC5629741 28775188

[5] Eibl JK, Gauthier G, Pellegrini D, et al. The effectiveness of telemedicine-delivered opioid agonist therapy in a supervised clinical setting. Drug Alcohol Depend 2017;176:133–138; doi: 10.1016/j.drugalcdep.2017.01.048 28535455

[6] Predmore ZS, Roth E, Breslau J, et al. Assessment of patient preferences for telehealth in post-COVID-19 pandemic health care. Vol. 4. JAMA Netw Open 2021;4(12):e2136405; doi: 10.1001/jamanetworkopen.2021.36405 34851400 PMC8637257

[7] Bajra R, Frazier W, Graves L, et al. Feasibility and Acceptability of a US National Telemedicine Curriculum for Medical Students and Residents: Multi-institutional Cross-sectional Study. JMIR Med Educ 2023;9:e43190; doi: 10.2196/43190 37155241 PMC10203924

[8] Curriculum Topics in Required and Elective Courses at Medical School Programs. AAMC. Available from: https://www.aamc.org/data-reports/curriculum-reports/data/curriculum-topics-required-and-elective-courses-medical-school-programs [Last accessed: Apr 21, 2023].

[9] Accreditation Council for Graduate Medical Education. ACGME-I Program Requirements for Graduate Medical Education in Family Medicine. 2023;1–63. Available from: http://www.acgme.org/acgmeweb/Portals/0/PFAssets/ProgramRequirements/120_family_medicine_07012014.pdf

[10] Palesy D, Forrest G, Crowley ME. Education guidelines, frameworks and resources for building virtual care capacity: An integrative review. Journal of Telemedicine and Telecare 2023;29(3):222–243. 36628539 10.1177/1357633X221149230

[11] Jortberg BT, Beck Dallaghan GL, Schatte D, et al. Expansion of telehealth curriculum: National survey of clinical education leaders. J Telemed Telecare 2022;28(6):464–468. 34775863 10.1177/1357633X211058330

[12] Khullar D, Mullangi S, Yu J, et al. The state of telehealth education at U.S. medical schools. Healthc (Amst) 2021;9(2):100522; doi: 10.1016/j.hjdsi.2021.100522 33548854

[13] Engelhart MD, Furst EJ, Hill WH, et al. Taxonomy of Educational Objectives: The Classification of Educational Goals Handbook1: Cognitive Domain [Internet]. Internet A. Bloom BB, ed. New York: Longman; 1956. pp. 186–193. https://archive.org/details/taxonomyofeducat0000bloo_o9o7/page/194/mode/2up

[14] Akman M, Wass V, Goodyear-Smith F., editors. How to Do Primary Care Research: A Practical Guide. 1st ed. CRC Press: Boca Raton; 2021: 232; doi: 10.1201/9781003110460

[15] McGaghie WC. Mastery learning: It is time for medical education to join the 21st century. Acad Med 2015;90(11):1438–1441. 26375269 10.1097/ACM.0000000000000911

